# Macrophage mannose receptor CD206 targeting of fluoride-18 labeled mannosylated dextran: A validation study in mice

**DOI:** 10.1007/s00259-024-06686-x

**Published:** 2024-03-27

**Authors:** Putri Andriana, Ruth Fair-Mäkelä, Heidi Liljenbäck, Salli Kärnä, Imran Iqbal, Konstantina Makrypidi, Johan Rajander, Ioannis Pirmettis, Xiang-Guo Li, Sirpa Jalkanen, Antti Saraste, Marko Salmi, Anne Roivainen

**Affiliations:** 1 Turku PET Centre, University of Turku, Kiinamyllynkatu 4-8, 20520 Turku, Finland; 2 Institute of Biomedicine, University of Turku, Turku, Finland; 3 InFLAMES Research Flagship Center, University of Turku, Turku, Finland; 4 Turku Center of Disease Modeling, University of Turku, Turku, Finland; 5 Institute of Nuclear and Radiological Science and Technology, Energy and Safety, NCSR “Demokritos”, Athens, Greece; 6 Turku PET Centre, Accelerator Laboratory, Åbo Akademi University, Turku, Finland; 7 Department of Chemistry, University of Turku, Turku, Finland; 8 MediCity Research Laboratory, University of Turku, Turku, Finland; 9 Turku PET Centre, Turku University Hospital, Turku, Finland; 10 Heart Center, Turku University Hospital and University of Turku, Turku, Finland

**Keywords:** Fluorine-18, Inflammation, Macrophage mannose receptor CD206, Mannosylated dextran, PET/CT

## Abstract

**Purpose:**

Aluminum fluoride-18-labeled 1,4,7-triazacyclononane-1,4,7-triacetic acid-conjugated mannosylated dextran derivative (Al[^18^F]F-NOTA-D10CM) is a new tracer for PET imaging. We report here on in vitro and in vivo validation of the tracer’s ability to target the macrophage mannose receptor CD206.

**Methods:**

First, the uptake of intravenously (i.v.) administered Al[^18^F]F-NOTA-D10CM was compared between wild-type (WT) and CD206^−/−^ knockout (KO) mice. C57BL/6N mice were injected with complete Freund’s adjuvant (CFA) in the left hind leg and the uptake of Al[^18^F]F-NOTA-D10CM after i.v. or intradermal (i.d.) injection was studied at 5 and 14 days after CFA induction of inflammation. Healthy C57BL/6N mice were studied as controls. Mice underwent PET/CT on consecutive days with [^18^F]FDG, i.v. Al[^18^F]F-NOTA-D10CM, and i.d. Al[^18^F]F-NOTA-D10CM. After the last imaging, Al[^18^F]F-NOTA-D10CM was i.v. injected for an ex vivo biodistribution study and autoradiography of inflamed tissues. Blood plasma samples were analyzed using high-performance liquid chromatography. To evaluate the specificity of Al[^18^F]F-NOTA-D10CM binding, an in vitro competitive displacement study was performed on inflamed tissue sections using autoradiography. CD206 expression was assessed by immunohistochemical staining.

**Results:**

Compared with WT mice, the uptake of Al[^18^F]F-NOTA-D10CM was significantly lower in several CD206^−/−^ KO mice tissues, including liver (SUV 8.21 ± 2.51 *vs.* 1.06 ± 0.16, *P* < 0.001) and bone marrow (SUV 1.63 ± 0.37 *vs.* 0.22 ± 0.05, *P* < 0.0001). The uptake of i.v. injected Al[^18^F]F-NOTA-D10CM was significantly higher in inflamed ankle joint (SUV 0.48 ± 0.13 *vs.* 0.18 ± 0.05, *P* < 0.0001) and inflamed foot pad skin (SUV 0.41 ± 0.10 *vs.* 0.04 ± 0.01, *P* < 0.0001) than in the corresponding tissues in healthy mice. The i.d.-injected Al[^18^F]F-NOTA-D10CM revealed differences between CFA-induced lymph node activation and lymph nodes in healthy mice. Ex vivo γ-counting, autoradiography, and immunohistochemistry supported the results, and a decrease of ~ 80% in the binding of Al[^18^F]F-NOTA-D10CM in the displacement study with excess NOTA-D10CM confirmed that tracer binding was specific. At 60 min after i.v. injection, an average 96.70% of plasma radioactivity was derived from intact Al[^18^F]F-NOTA-D10CM, indicating good in vivo stability. The uptake of Al[^18^F]F-NOTA-D10CM into inflamed tissues was positively associated with the area percentage of CD206-positive staining.

**Conclusion:**

The uptake of mannosylated dextran derivative Al[^18^F]F-NOTA-D10CM correlated with CD206 expression and the tracer appears promising for inflammation imaging.

**Graphical abstract:**

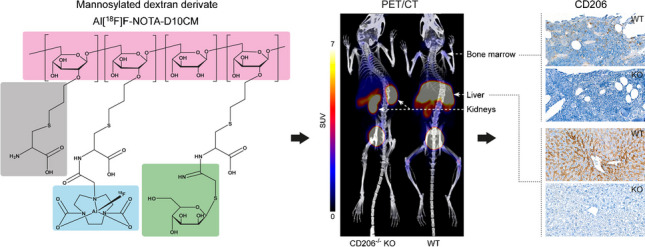

**Supplementary information:**

The online version contains supplementary material available at 10.1007/s00259-024-06686-x.

## Introduction

The macrophage mannose receptor cluster of differentiation 206 (CD206) is a 175 kDa C-type lectin transmembrane protein [1]. It belongs to the mannose receptor family, which also includes recycling endocytic receptor Endo180 (CD280), M-type phospholipase A_2_ receptor, and dendritic and thymic epithelial cell-205 receptor DEC-205 (CD205) [2]. These share a similar extracellular domain structure consisting of a C-type lectin-like domain (CTLD), a fibronectin type II domain, and an NH_2_-terminal cysteine-rich domain. However, each member has distinct ligand recognition properties and a cell-type specific expression pattern. CD206 and Endo180 are the only family members with sugar-binding properties, including binding to mannose via CTLD4 in CD206 and via CTLD2 in Endo180. Nevertheless, CD206 is the only member of the mannose receptor family with a functional cysteine-rich domain that binds to glycoproteins and sulfated sugars, including glycosylated mannose [1].

The presence of the macrophage mannose receptor CD206 in lymph nodes and both afferent and efferent lymphatics [3, 4] indicates that it has an immune signaling role during the inflammation process. This function of CD206 has been used for sentinel lymph node mapping by noninvasive imaging [5]. In addition, since CD206 is predominantly expressed on the surface of alternatively activated (M2-type) macrophages [6] associated with the inflammation resolution process, targeting of CD206 may allow noninvasive visualization and quantification of inflammation, and assessment of CD206 expression by molecular imaging might be useful for monitoring disease activity and treatment efficacy.

Technetium-99m radiolabeled mannosylated dextran (^99m^Tc-Tilmanocept) was the first U.S. Food and Drug Administration (FDA)-approved imaging agent for use as an intradermal (i.d.) injection for sentinel lymph node mapping with single-photon emission computed tomography (SPECT) [5]. Another mannosylated dextran, ^99m^Tc(CO)_3_–DCM20, was developed by Pirmettis and co-workers and evaluated in mice [7]. In addition, several non-mannosylated dextran conjugates for targeting of CD206 have been reported, including a ^68^Ga-radiolabeled nanobody against macrophage mannose receptor (^68^Ga-NOTA-anti-MMR Nb) [8, 9], radiolabeled 2-deoxy-2-[^18^F]fluoro-*D*-mannose (^18^F-FDM) [10], and ^68^Ga-radiolabeled mannosylated human serum albumin (^68^Ga-NOTA-MSA) [11, 12]. An example of another type of CD206-targeted compounds is the recently introduced new ^68^Ga-labeled peptide RP832c, which has been applied for PET imaging of tumor-associated macrophages [13]. Although CD206-targeted PET imaging agents have been utilized in various studies, there is still room for new tracer developments to find the most optimal one for clinical applications.

We recently described the new positron emission tomography (PET) tracer “aluminum fluoride-18-labeled 1,4,7-triazacyclononane-1,4,7-triacetic acid-conjugated mannosylated dextran derivative Al[^18^F]F-NOTA-D10CM (22 kDa)”, which contains a dextran–cysteine–mannose glycoprotein structure and is aimed at targeting of the macrophage mannose receptor CD206 [14]. To evaluate Al[^18^F]F-NOTA-D10CM as a PET tracer for inflammation, we assessed its ability to specifically target macrophage mannose receptor CD206 by comparing its distribution between CD206^−/−^ knockout (KO) and wild-type (WT) mice. Furthermore, we studied its uptake in inflamed skin and lymph nodes draining the inflammatory site after intravenous (i.v.) and i.d. administration in mice with complete Freund’s adjuvant (CFA)-induced inflammation.

## Materials and methods

### Animals and experimental design

The CD206^−/−^ deficient (referred to as CD206^−/−^ KO) MB6.129P2-*Mrc1*^tm1Mnz/J^ mouse was previously described [3, 4], and was studied together with age- and sex-matched WT littermate controls in this work. Female C57BL/6N mice were purchased from Janvier Labs for CFA induction studies. Mice were housed under controlled environmental conditions with a 12:12 h light:dark cycle at the Central Animal Laboratory of the University of Turku.

To induce inflammation, a single 20 µL volume of a mixture containing CFA (F5881, Sigma Aldrich) and 2 µg of ovalbumin (vac-pova, InvivoGen) was injected subcutaneously into the dorsal side of the left hind paws of C57BL/6N mice using Microfine Demi 0.3 mL syringes (BD) and a 30-G needle. The mice were studied on day 5 or day 14 after CFA induction (day 0). Non-inflamed C57BL/6N mice were used as controls.

**Fig. 1:**
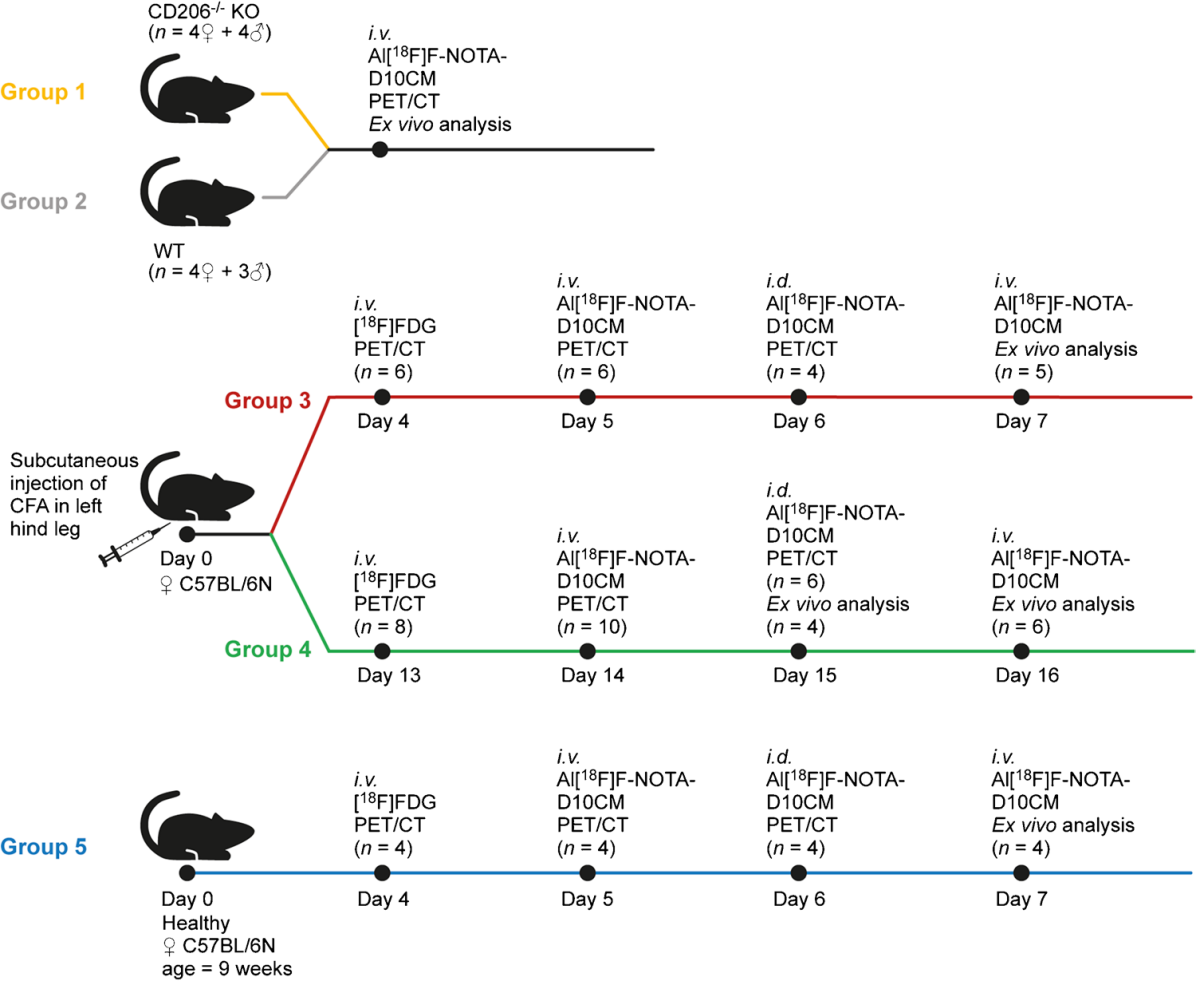
Study design

The experimental study design is shown in Fig. 1. A total of 35 mice were divided into five groups: Group 1: CD206^−/−^ KO (*n* = 4 females + 4 males, weight 25.16 ± 6.03 g, age 10–15 weeks); Group 2: WT (*n* = 4 females + 3 males, 26.72 ± 3.52 g, 10–23 weeks); Group 3: day 5 after CFA induction (*n* = 6 females, 20.20 ± 1.69 g, 9 − 10 weeks); Group 4: day 14 after CFA induction (*n* = 10 females, 20.09 ± 1.22 g, 11 − 12 weeks); and Group 5: healthy controls (*n* = 4 females, 20.60 ± 0.50 g, 9 weeks). The CFA inductions studies were only performed in WT mice. The mice underwent PET/computed tomography (CT) on consecutive days after i.v. injection of [^18^F]FDG (4.95 ± 0.51 MBq) or Al[^18^F]F-NOTA-D10CM (5.66 ± 2.18 MBq [range: 3.41–10.00 MBq], 12.45 ± 4.80 µg [range: 7.50–22.00 µg], 0.57 ± 0.22 nmol [range: 0.34–1.00 nmol]), and i.d. injection of Al[^18^F]F-NOTA-D10CM into the left hind paw (4.55 ± 1.69 MBq [range: 2.39–9.96 MBq], 10.00 ± 3.73 µg [range: 5.26 − 21.91 µg], 0.45 ± 0.16 nmol [range: 0.24–1.00 nmol]). On the last day of the study, the mice were i.v. injected with Al[^18^F]F-NOTA-D10CM (7.55 ± 2.84 MBq) and ex vivo analyses were performed 60 min post-injection.

All animal experiments were approved by the national Project Authorization Board in Finland (license numbers ESAVI/8648/2020 and ESAVI/14685/2020) and were carried out in compliance with EU Directive 2010/EU/63 on the protection of animals used for scientific purposes.

### Radiosynthesis of Al[^18^F]F-NOTA-D10CM

A1[^18^F]F-NOTA-D10CM was prepared according to a previously published method [14]. Briefly, NOTA-D10CM (6.8 nmol in 50 µL water) was radiolabeled with [^18^F]fluoride (220 µL in saline) by heating at 100°C for 13 min in a mixture of A1C1_3_ in 1 M sodium acetate buffer (pH 4.0, 40 µL), acetonitrile (60 µL), and 150 mM ascorbic acid (40 µL), with 0.1% trifluoroacetic acid (TFA) in water (810 µL) then being added after the reaction mixture was cooled to 40°C. The product was purified with radiodetector-coupled high-performance liquid chromatography (radio-HPLC) using a semipreparative C18 Jupiter Proteo column (250  ×10 mm, 4 µm, 90 Å; Phenomenex) with a gradient of 0.1% TFA in water (solvent A) and 0.1% TFA in acetonitrile (solvent B). The A1[^18^F]F-NOTA-D10CM was collected in an end product bottle containing 15 mM ascorbic acid in phosphate-buffered saline (PBS).

### In vivo PET/CT

Mice were imaged with PET and CT systems (Molecubes) under isoflurane anesthesia (4 − 5% induction, 1.5 − 2% maintenance). The tail vein was cannulated before imaging. CT was performed for attenuation correction and anatomical reference. A 20 min static PET acquisition was performed 90 min post-injection of [^18^F]FDG. With Al[^18^F]F-NOTA-D10CM, a 120 min dynamic PET acquisition was started at the time of injection. PET data obtained in a list-mode were reconstructed into 10 × 60 s, 4 × 300 s, and 9 × 600 s time frames using a three-dimensional ordered subsets expectation maximization algorithm. PET/CT images were analyzed using Carimas 2.10 software (www.turkupetcentre.fi/carimas/). Regions of interest (ROIs) were defined manually on the main organs using CT as the anatomical reference. The inflamed skin area was confirmed by [^18^F]FDG uptake (Supplementary Fig. 1). At least three consecutive planes at 50 − 60 min after Al[^18^F]F-NOTA-D10CM injection were used for quantitative analysis. Time-activity curves of standardized uptake value (SUV) as a function of time post-injection were extracted from dynamic PET data.

### Ex vivo biodistribution

Mice were sacrificed by cardiac puncture and cervical dislocation under isoflurane anesthesia at 60 min after the last i.v. Al[^18^F]F-NOTA-D10CM injection. Tissues of interest were excised and weighed, and their radioactivities were measured with a γ-counter (Triathler 3 ˝, Hidex). The results were decay-corrected to the time of injection, compensated for radioactivity remaining in the tail, and expressed as a percentage of the injected radioactivity dose per gram of tissue (%ID/g).

### Ex vivo digital autoradiography

The left-side inflamed foot pad skin and the left inflamed popliteal lymph node of the mice with CFA-induced inflammation and corresponding tissues from healthy control mice were collected for cryosectioning. The samples were embedded and frozen in Tissue-Tek O.C.T. Compound (Sakura), cut into three serial 20 µm and five 6 µm sections, and collected onto microscopic slides. The slides were briefly air-dried, opposed to phosphor imaging plates (BAS-TR2025, Fuji), and exposed overnight, and the plates were scanned with Fuji Analyzer BAS-5000. Following autoradiography, frozen sections were stained with hematoxylin–eosin (H&E) for histological reference or were used for CD206 detection. ROIs were analyzed on superimposed autoradiography and digitalized H&E images using Carimas software. The results are expressed as photostimulated luminescence per square millimeter (PSL/mm^2^), decay-corrected for injection time and exposure time, and normalized for the injected radioactivity dose. The target-to-background ratio (TBR) was determined from inflamed and non-inflamed areas defined according to the detection of CD206 (CD206_high_ area/CD206_low_ area). CD206_high_/CD206_low_ areas were defined on the basis of histology and immunohistochemical and immunofluorescence staining of consecutive sections.

### In vivo stability of Al[^18^F]F-NOTA-D10CM

A subset of healthy C57BL/6N mice (*n* = 6 females, 19.77 ± 0.71 g, 8 − 9 weeks) were i.v. injected with A1[^18^F]F-NOTA-D10CM (9.76 ± 0.43 MBq [range: 9.07–10.62 MBq], 19.09 ± 7.22 µg [range: 19.95–23.36 µg], 0.87 ± 0.33 nmo1 [range: 0.91–1.06 nmo1]). Blood was withdrawn by cardiac or saphenous vein puncture, and collected into heparinized tubes at 5 min (600.00 ± 200.00 µL, *n* = 3), 10 min (600.00 ± 200.00 µL, *n* = 3), 20 min (160.00 ± 17.32 µL, *n* = 3), 40 min (450.00 ± 259.81 µL, *n* = 3), and 60 min (533.33 ± 115.47 µL, *n* = 3) post-injection. Plasma was separated by centrifugation (14,000 × *g* for 5 min at 4°C), and then plasma proteins were precipitated with 10% sulfosalicylic acid and separated by centrifugation (14,000 × *g* for 2 min at room temperature). The plasma supernatant was filtered through a 0.45 µm Minispike filter (Waters), and diluted with 0.1% TFA in water, and analyzed with radio-HPLC using a C18 Jupiter Proteo semipreparative column (250 × 10 mm, 5 µm, 90 Å; Phenomenex) and a gradient of 0.1% TFA in water (solvent A) and 0.1% TFA in acetonitrile (solvent B).

### In vitro competitive displacement assay

Cryosections of inflamed popliteal lymph node and foot pad skin (6 µm thickness) from mice with CFA-induced inflammation were defrosted at 4 °C for 40 min. The sections were pre-incubated in 2-[4-(2-hydroxyethyl)piperazin-1-yl]ethanesulfonic acid buffer (HEPES, Sigma Aldrich) (pH 7.4) containing 10 mM Ca^2+^ for 15 min at room temperature in an incubation chamber. For the total binding study, slides were transferred to another chamber containing Al[^18^F]F-NOTA-D10CM (23 kBq/mL) in a buffer. For the competitive binding assay, adjacent tissue sections were first incubated with Al[^18^F]F-NOTA-D10CM (23 kBq/mL) and a 400-fold molar excess of unlabeled NOTA-D10CM in a buffer for 70 min. Then, the slides were rinsed twice with a cold buffer and dipped into cold water. The slides were briefly air-dried, exposed overnight to phosphor imaging plates, scanned, and analyzed as described above. Experiments were performed in triplicate using tissue samples from three mice (*n* = 3).

### Histology, immunohistochemistry, and immunofluorescence

Following autoradiography, frozen sections of foot pad skin and popliteal lymph nodes were stained with H&E (20 µm) for histological reference or were used for CD206 detection (6 µm).

For CD206 immunohistochemical staining [14], sections were fixed in 4% paraformaldehyde then submitted to antigen retrieval, washing, and blocking of endogenous peroxidase activity. The sections were then incubated for 60 min at room temperature with polyclonal rabbit anti-mannose receptor (CD206/MRC1) antibody (working dilution 1:10,000; ab64693, Abcam), rinsed, and incubated with the secondary antibody (BrightVision horseradish peroxidase conjugated goat anti-mouse IgG, DPVR110HRP; WellMed) for 30 min at room temperature. The sections were reacted with 3,3-diaminobenzidine (BrightDAB, BSo4-110; WellMed), counterstained with Mayer’s hematoxylin, mounted with Pertex, and dried overnight.

For immunofluorescence staining, sections were first fixed with ice-cold acetone for 3 min, and then incubated for 60 min with Alexa Fluor® 488 monoclonal rat anti-mouse CD206 antibody (BioLegend® 14170, clone C068C2, working dilution 10 µg/mL), Alexa Fluor® 594 monoclonal rat anti-mouse CD31 antibody (BioLegend® 102520, clone MEC13.3, working dilution 10 µg/mL), and Alexa Fluor® 647 monoclonal rat anti-mouse CD11b antibody (BioLegend® 101220, clone M1/70, working dilution 10 µg/mL) in a humidified chamber covered to prevent light exposure. The sections were then rinsed twice with PBS and counter stained with 4′,6-diamidino-2-phenylindole (DAPI, Invitrogen D1306). The slides were then washed twice with PBS and coverslips were mounted with ProLong Gold Antifade (Invitrogen P36930) mounting medium.

Stained sections were scanned with a digital slide scanner (Pannoramic P1000 or Pannoramic 250 Flash, 3DHISTECH Ltd.) and examined using Pannoramic Viewer 1.15 software (3DHISTECH Ltd.). For quantitative analysis of CD206 area-%, sections were classified as CD206_high_ and CD206_low_ areas by fuzzy selection in GIMP (version 2.10.24), based on a specific RGB color threshold of 19 with H&E used as a reference (Supplementary Fig. 2). Quantification of CD206_high_ and CD206_low_ area-% was performed by color deconvolution analysis based on hematoxylin and DAB stains using ImageJ 1.52n software Fiji (Wayne Rasband, https://imagej.net/software/fiji/).

Immunofluorescently stained sections were imaged with the 3i Spinning Disk confocal microscope (Intelligent Imaging Innovations) with a Plan-Apochromat 20 × /0.8 objective. Z stack images were acquired with SlideBook 6 software (Intelligent Imaging Innovations). ImageJ software was used to create maximum intensity projections and perform background subtractions and linear brightness adjustments.

### Statistical analysis

Results are expressed as mean ± standard deviation (SD). Differences between groups were analyzed with unpaired Student’s *t* tests or one-way analysis of variance (ANOVA). *P*-values < 0.05 are considered statistically significant and referred to as *P < 0.05, **P < 0.01, and ***P < 0.001. Associations between two variables were evaluated using Pearson’s correlation coefficient.

## Results

### Radiosynthesis and stability of Al[^18^F]F-NOTA-D10CM

The Al[^18^F]F-NOTA-D10CM was produced with a molar activity of 9.87 ± 4.59 GBq/µmol (*n* = 4) and radiochemical purity of ≥ 95% at the end of synthesis. The decay-corrected radioactivity yield was 1156.14 ± 440.94 MBq (19.89% ± 7.02), with a radioactivity concentration of 472.41 ± 218.73 MBq/mL at the end of synthesis (*n* = 15). The radio-HPLC analysis of blood plasma samples from healthy mice showed 98.90% ± 1.90 (*n* = 3), 97.83% ± 3.76 (*n* = 3), 99.13% ± 1.51 (*n* = 3), 96.42% ± 2.47 (*n* = 3), and 91.24% ± 1.07 (*n* = 3) intact Al[^18^F]F-NOTA-D10CM at 5, 10, 20, 40, and 60 min after i.v. injection, respectively (Supplementary Fig. 3).

### Intravenous Al[^18^F]F-NOTA-D10CM in WT mice versus CD206^−/−^ KO mice

**Fig. 2:**
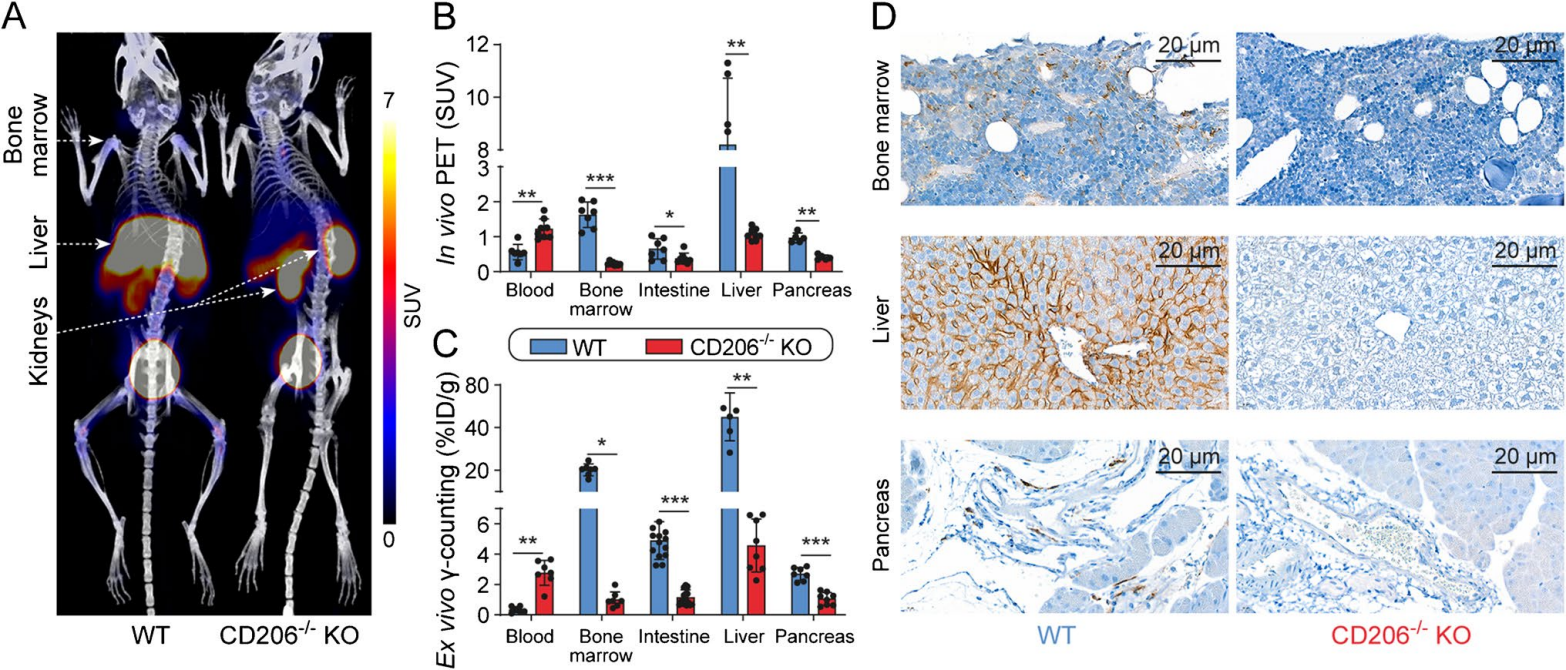
**A** Representative coronal maximum intensity projection images of Al[^18^F]F-NOTA-D10CM PET/CT of wild-type (WT) and CD206^−/−^ knockout (KO) mice. The difference in radioactivity concentration after intravenous injection is most prominent in the liver. Quantification of Al[^18^F]F-NOTA-D10CM uptake in tissues of interest by (**B**) in vivo PET and (**C**) ex vivo γ-counting at 2 h post-injection. Bars represent mean values and error bars are standard deviations. *P < 0.05, **P < 0.01, ***P < 0.001. **D** CD206 immunohistochemical staining shows abundant positivity in the bone marrow, liver, and pancreas of WT mice, whereas no specific staining is seen in CD206^−/−^ KO mice

On in vivo Al[^18^F]F-NOTA-D10CM PET/CT, the highest SUVs were found in the liver (8.21 ± 2.51), spleen (3.56 ± 0.85), kidneys (1.94 ± 0.93), salivary gland (1.86 ± 0.34), and bone marrow (1.63 ± 0.37) of the WT mice. Compared with WT mice, uptake in CD206^−/−^ KO mice was significantly lower in the liver (1.06 ± 0.16, *P* < 0.001) and bone marrow (0.22 ± 0.05, *P* < 0.0001) (Fig. 2, Supplementary Fig. 4). These results were supported by the ex vivo γ-counting (Supplementary Table 1). The radioactivity concentration was higher in the blood of CD206^−/−^ KO mice (1.22 ± 0.29) than in the blood of WT mice (0.56 ± 0.22, *P* < 0.001). Both in vivo and ex vivo results revealed significantly higher uptake of radioactivity in the spleen (a blood-rich organ reflecting blood radioactivity) and kidneys (excretion route of unbound radioactivity) in CD206^−/−^ KO mice than in WT mice.

### Intravenous Al[^18^F]F-NOTA-D10CM in mice with CFA-induced inflammation

Experiments in mice with CFA-induced inflammation and healthy control mice included both i.d. and i.v. injection routes of Al[^18^F]F-NOTA-D10CM at day 5 and day 14 after CFA induction. Mice were visually screened for a swollen foot pad using [^18^F]FDG PET/CT a day before Al[^18^F]F-NOTA-D10CM PET/CT. Quantitative analyses were performed on inflamed tissue that was histologically confirmed by H&E staining.

**Fig. 3:**
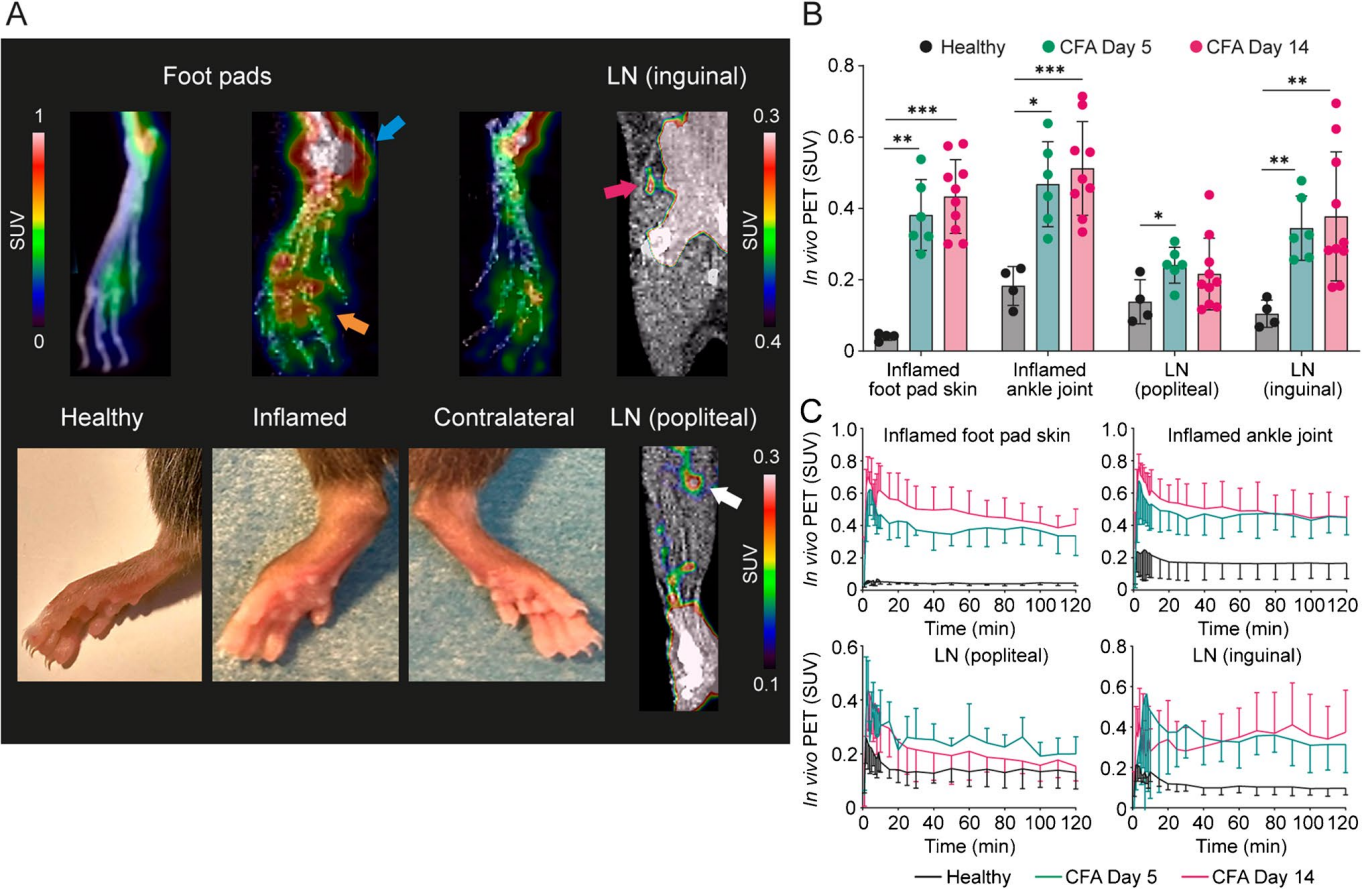
**A** Representative maximum intensity projection PET/CT images of Al[^18^F]F-NOTA-D10CM intravenously administered into the hind legs of a healthy mouse and a mouse on day 5 after complete Freund’s adjuvant (CFA) inflammation induction, and coronal views of inflamed lymph nodes (LN). In the photographs, the inflamed hind leg appears clearly swollen. The blue arrow denotes the ankle joint, the yellow arrow denotes the foot pad, and the red and white arrows denote LNs. **B** Quantification of Al[^18^F]F-NOTA-D10CM uptake by in vivo PET 50 − 60 min post-injection as standardized uptake value (SUV). *P < 0.05, **P < 0.01, ***P < 0.001. (**C**) Time-activity curves for Al[^18^F]F-NOTA-D10CM uptake. Bars and curves represent means, and error bars are standard deviation

The in vivo PET/CT of mice that were i.v.-injected with Al[^18^F]F-NOTA-D10CM (Fig. 3, Supplementary Figs. 5 and 6) showed significantly higher SUVs in the inflamed left ankle joint (0.48 ± 0.13 *vs.* 0.18 ± 0.05, *P* < 0.0001), foot pad skin (0.41 ± 0.10 *vs.* 0.04 ± 0.01, *P* < 0.0001), popliteal lymph node (0.24 ± 0.05 *vs.* 0.14 ± 0.06, *P* < 0.0001), and inguinal lymph node (0.37 ± 0.15 *vs.* 0.10 ± 0.04, *P* = 0.01) in comparison with the controls. These results were supported by the ex vivo γ-counting (Supplementary Table 2). There were no statistically significant differences in tracer uptake between day 5 and day 14 after CFA induction. The pooled results from these 2 days showed the highest SUVs in liver (8.44 ± 1.12), spleen (4.46 ± 0.91), and bone marrow (2.52 ± 0.62), with the values being significantly higher than in control mice.

**Fig. 4:**
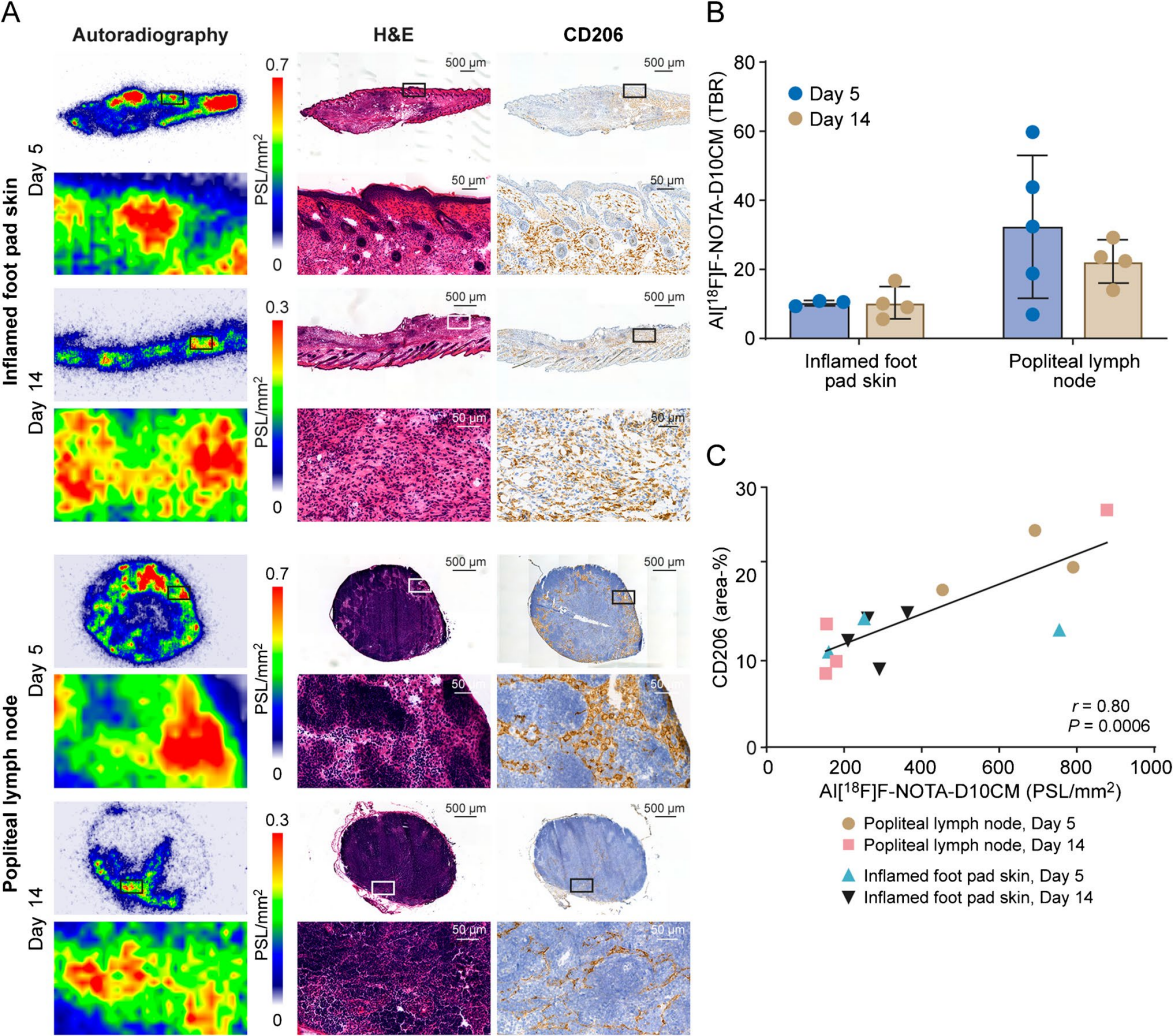
**A** Representative Al[^18^F]F-NOTA-D10CM autoradiographs after intravenous injection, hematoxylin–eosin (H&E) staining, and CD206 immunohistochemical staining of inflamed foot pad skin (upper panel) and inflamed popliteal lymph node (lower panel) at 5 days and 14 days after complete Freund’s adjuvant-induced inflammation. The high focal Al[^18^F]F-NOTA-D10CM uptake co-localizes with inflammatory foci seen in H&E and CD206-positive staining (in brown). PSL, photostimulated luminescence. **B** Target-to-background ratio derived from Al[^18^F]F-NOTA-D10CM autoradiographs as CD206_high_ area/CD206_low_ area. (**C**) Correlation between Al[^18^F]F-NOTA-D10CM uptake and CD206 area-%

Ex vivo digital autoradiography of inflamed foot pad skin and inflamed popliteal lymph node showed co-localization of Al[^18^F]F-NOTA-D10CM uptake with CD206-positivity according to immunohistochemical staining in adjacent tissue sections. The target-to-background ratio (CD206_high_ area/CD206_low_ area) of Al[^18^F]F-NOTA-D10CM in the inflamed tissues was high on both day 5 (skin 10.24 ± 0.79, lymph node 32.32 ± 20.68) and day 14 (skin 10.36 ± 4.66, lymph node 22.29 ± 6.27) after CFA induction (Fig. 4, Supplementary Fig. 7). In addition, there was a positive correlation between the uptake of Al[^18^F]F-NOTA-D10CM by autoradiography and the area percentage of CD206-positive staining (*r* = 0.80, *P* < 0.001, Fig. 4C).

**Fig. 5:**
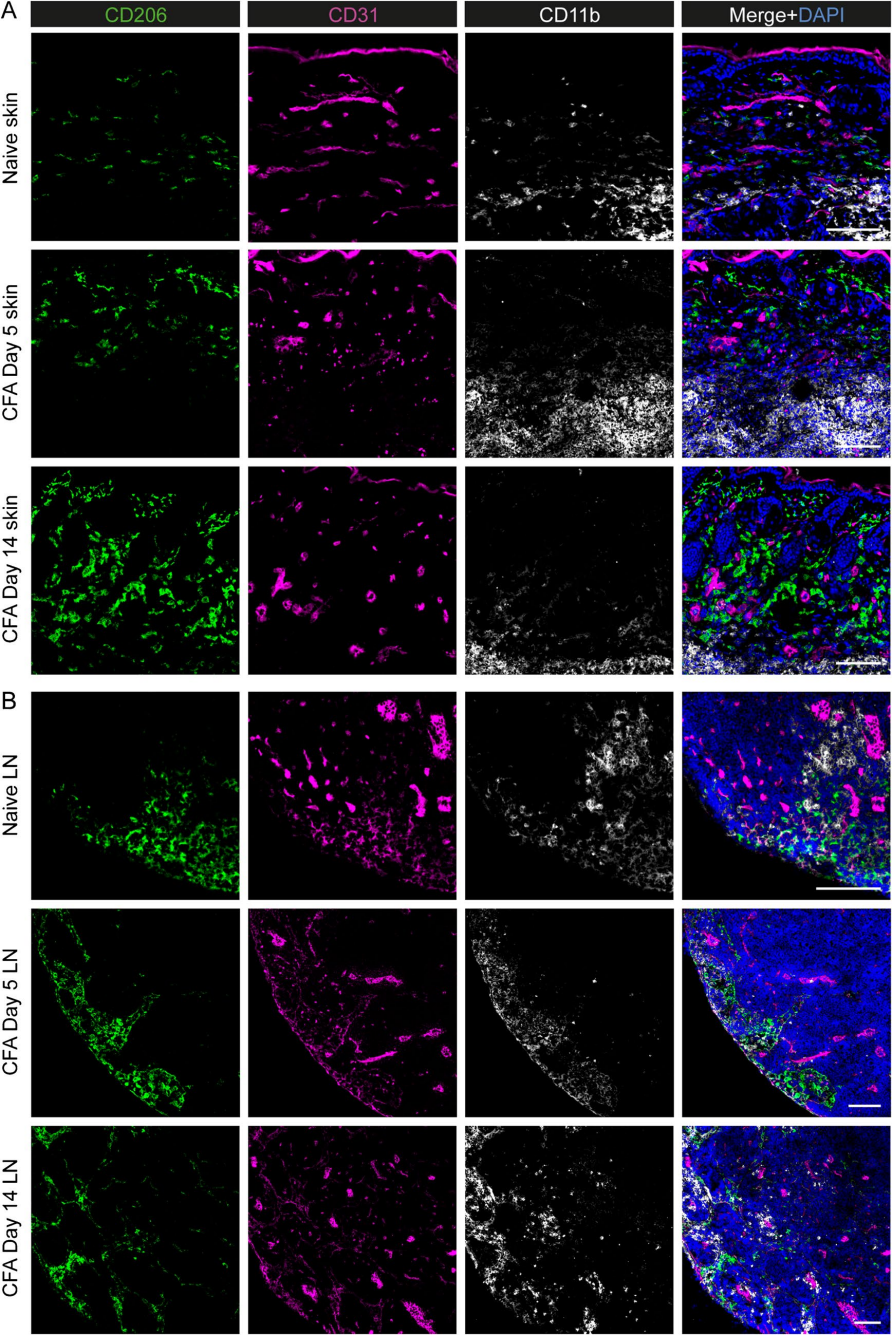
Representative immunofluorescence staining of healthy and inflamed foot pad skin (**A**) shows an increase in CD206^+^ macrophages in the dermis at days 5 and 14 after induction with complete Freund’s adjuvant (CFA). CD31 labels blood and lymphatic vessels in the dermis (positive staining of epidermis is nonspecific), and CD11b labels myeloid cells. **B** In the draining popliteal lymph node (LN), CD206 is expressed in the CD31^+^ medullary sinus in both healthy and inflamed lymph nodes. Scale bars are 100 µm

The identity of CD206^+^ cells in the CFA induction samples was determined using immunofluorescence staining of CD206, CD31 (expressed by blood and lymphatic endothelial cells), and CD11b (expressed by myeloid cells). The images showed an increase in CD206 signal in the footpad skin samples upon CFA induction, which was most likely due to skin-infiltrating CD206^+^ macrophages. In lymph nodes, CD206 was mainly expressed by lymphatic endothelial cells in the medullary sinus (CD206^+^ and CD31^+^ double-positive cells), which undergo morphological changes during inflammation. CD206^+^ and CD11b^+^ double-positive macrophages were also found in the medullary sinus (Fig. 5).

### Intradermal Al[^18^F]F-NOTA-D10CM in mice with CFA-induced inflammation

**Fig. 6:**
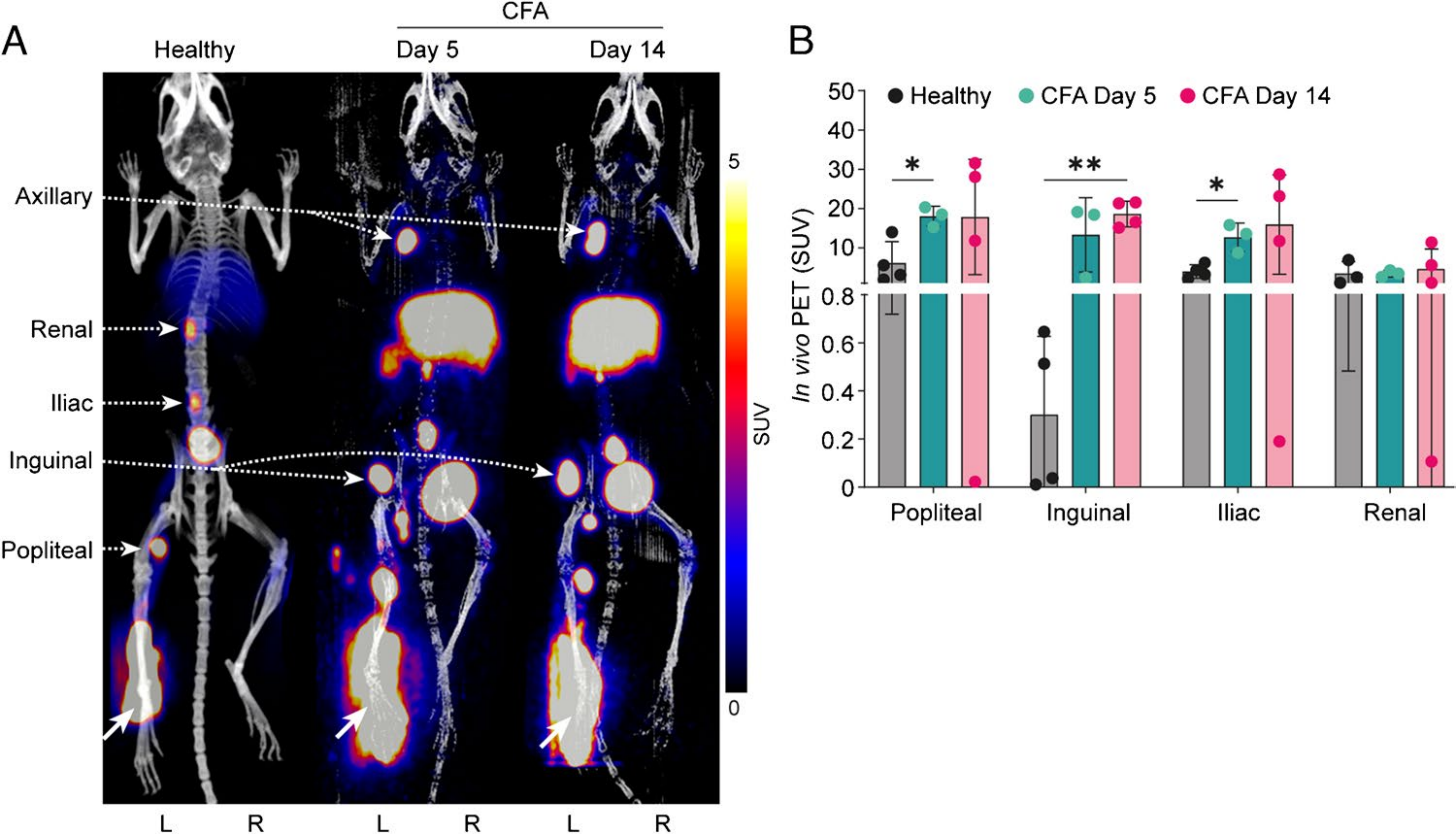
**A** In vivo PET/CT after intradermal injection of Al[^18^F]F-NOTA-D10CM (white arrows) reveals a change in the lymphatic drainage pathway after induction of inflammation by complete Freund’s adjuvant (CFA). **B** In healthy mice (*n* = 4), the tracer was distributed from the popliteal lymph node to the iliac and renal lymph nodes, whereas the route to the inguinal lymph node was low. Five days after CFA induction, the tracer was distributed from the popliteal lymph node to the inguinal lymph node, with the route to the iliac lymph node being increased. Fourteen days after CFA induction, the tracer’s route to the inguinal lymph node was significantly increased (*P* = 0.002). We also noted uptake of the tracer in inflamed axillary lymph node. Bars represent standardized uptake values (SUV) at 2 h after intradermal Al[.^18^F]F-NOTA-D10CM injection and error bars are standard deviations. *P < 0.05, **P < 0.01

In healthy mice, the i.d. administered Al[^18^F]F-NOTA-D10CM first passed to the draining popliteal lymph node (SUV 6.16 ± 5.44), then to the iliac lymph node (SUV 3.99 ± 1.74), and finally to the renal lymph node (SUV 3.51 ± 3.03) (Fig. 6). In the mice with CFA-induced inflammation, an alternative lymphatic route [15] was active; at day 5 after CFA induction, Al[^18^F]F-NOTA-D10CM was distributed from the injection site to the popliteal lymph node (SUV 18.04 ± 2.55) and inguinal lymph node (SUV 13.34 ± 9.44), and the route to the iliac lymph node and renal lymph node was still active. At day 14 after CFA induction, the alternative lymphatic pathway was significantly more active than in healthy controls and the CFA day 5 group. The ex vivo γ-counting confirmed the PET/CT results (Supplementary Table 3).

### Binding specificity of Al[^18^F]F-NOTA-D10CM

**Fig. 7:**
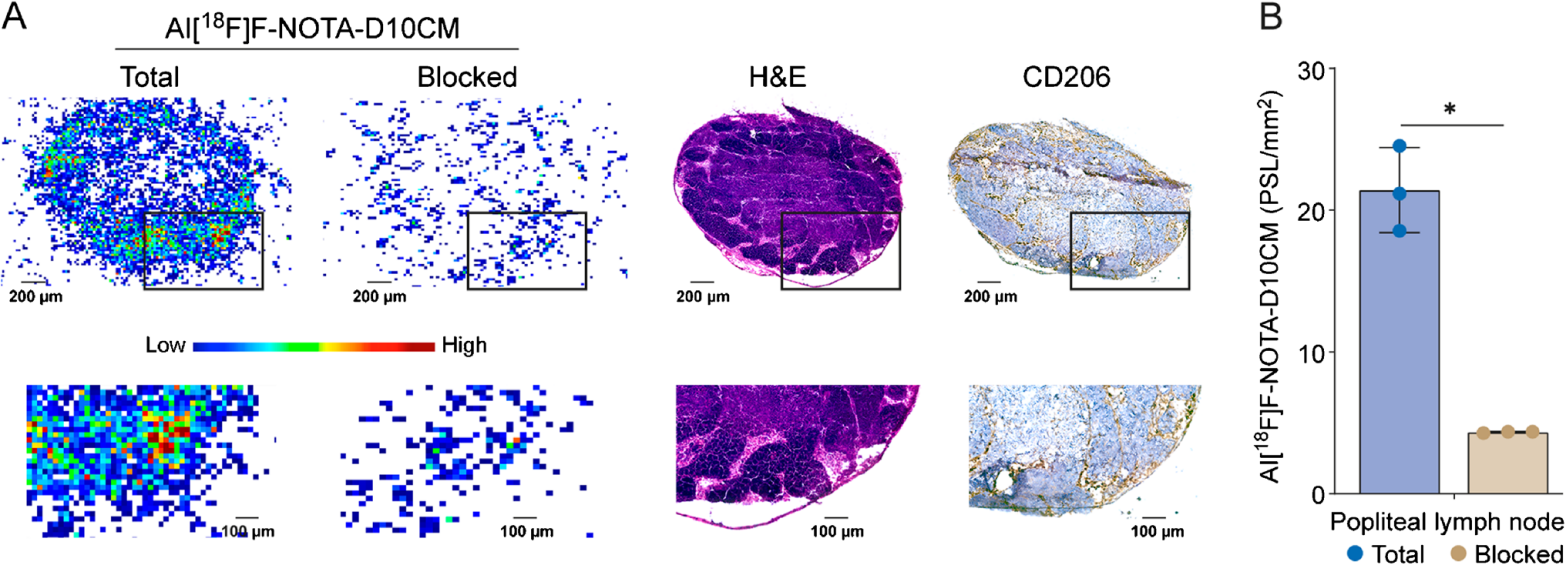
**A** Representative digital autoradiographs from the in vitro competitive displacement assay on adjacent sections of an inflamed popliteal lymph node confirmed by H&E and CD206 immunohistochemical staining. Positive CD206 staining is shown as a brown color. **B** Co-incubation with excess of unlabeled NOTA-D10CM significantly reduced the binding of Al[^18^F]F-NOTA-D10CM by 79.48% ± 2.63. *P < 0.05

The in vitro competitive displacement assay performed on cryosections from mice with CFA-induced inflammation revealed that co-incubation with an excess of unlabeled NOTA-D10CM reduced the binding of Al[^18^F]F-NOTA-D10CM by 79.48% ± 2.63 in inflamed popliteal lymph node and 40.70% ± 19.93 in inflamed foot pad skin (Fig. 7).

## Discussion

In this study, we confirmed macrophage mannose receptor CD206 targeting by the new mannosylated dextran derivative Al[^18^F]F-NOTA-D10CM. First, comparative studies in WT mice and CD206^−/−^ KO mice revealed significant differences in tracer biodistribution. Then, we found that i.v. and i.d. administered Al[^18^F]F-NOTA-D10CM specifically targeted overexpression of macrophage mannose receptor CD206 during skin inflammation and associated lymph node activation. These results demonstrate that Al[^18^F]F-NOTA-D10CM PET enables in vivo evaluation of CD206 expression, and pave the way for further translational studies in experimental disease models.

### CD206^−/−^ KO and inflammatory mouse models

CD206 expression is well characterized in mouse tissues, being present on several different cell types, including macrophages, lymphatic endothelial cells, and sinusoidal liver endothelial cells. CD206 is known to take part in antigen clearance and to interact with several microbial products through its CTLD domain, yet CD206 deficiency does not result in increased susceptibility to infections in mice [1]. Instead, CD206 may take part in antigen processing and presentation, leading to abrogated humoral responses [16]. For these studies, the availability of CD206^−/−^ KO mice provided a particularly elegant specificity control for Al[^18^F]F-NOTA-D10CM.

CFA has been widely used to augment adaptive immune responses and chronic inflammation in the context of experimental antibody production and autoimmune disease modeling (such as adjuvant-induced arthritis) [17, 18]. After subcutaneous administration, the adjuvant induces inflammation both locally at the injection site and in the chain of draining lymph nodes. In our study, we were able to observe increased uptake of i.v. and i.d. administered Al[^18^F]F-NOTA-D10CM in studied tissues at days 5 and 14 after CFA induction. The increased uptake of the tracer in ankle joint confirms that CFA induces joint inflammation in mice. As expected, tracer uptake in draining popliteal lymph node was high because of the ongoing immune response. Moreover, we also detected tracer uptake in the inguinal lymph node after i.d. administration, suggesting that this alternative lymphatic drainage pathway is activated upon CFA induction [15]. In conclusion, the uptake of Al[^18^F]F-NOTA-D10CM in CD206^−/−^ KO mice, its correlation with the CD206 induction pattern upon inflammation, and the in vitro competition studies together indicate that the tracer predominantly binds to the CD206 target molecule, although additional target molecules may contribute to the overall uptake.

### Comparison with previous studies on mannosylated dextran derivatives

The radiolabeled mannosylated dextran derivative ^99m^Tc-Tilmanocept (Lymphoseek®, 23 amino acids, 55 mannose moieties, and 8 diethylenetriaminepentaacetic acid [DTPA] chelators per dextran molecule), which binds to macrophage mannose receptor CD206, was approved by the FDA in 2014 for lymphatic mapping and lymph node localization in breast cancer, melanoma, squamous cell carcinoma, and other solid tumors [19]. Lymphoseek® has also been ^18^F-labeled through covalent bond formation, but this resulted in a low molar activity of 1.85 GBq/µmol [20]. Pirmettis and co-workers developed the new mannosylated dextran DCM20 (6 cysteines and 24 mannoses) bearing an S-derivatized cysteine chelator, which is a more ideal chelator for ^99m^Tc stabilization than the DTPA used in ^99m^Tc-Tilmanocept. In mice, ^99m^Tc(CO)_3_–DCM20 shows rapid clearance and high uptake and retention in the sentinel lymph nodes, but it has not yet been evaluated in humans [7]. Further studies by Papassava and co-workers compared different molecular sizes between D10CM and D500CM (MW 21.2–805.6 kDa, 7–142 cysteines, 19–645 mannoses) for the detection of sentinel lymph nodes, and concluded that ^99m^Tc(CO)_3_-D75CM (MW 111.24 kDa, 23 cysteines, 74 mannoses) was superior, with accumulation twice that of ^99m^Tc(CO)_3_-DCM20 [21].

In our study, we preferred the smaller size of D10CM (21.3 kDa, 7 cysteines, 19 mannoses) for Al[^18^F]F-radiolabeling, with it resulting in good molar activity of 10 GBq/µmol, which helped in revealing the tracer’s potential for imaging of inflammation [14]. While most radiolabeled mannosylated dextrans were developed for lymph node mapping, we evaluated Al[^18^F]F-NOTA-D10CM for PET imaging of inflammation. For inflammation imaging, we investigated both i.v. and i.d. routes of tracer administration, and found the latter to be more specific for imaging of lymphatics and lymph node activation.

### Comparison with other macrophage mannose receptor-targeted radiopharmaceuticals

Mannosylated dextran is not the only conjugate developed for macrophage mannose receptor CD206 targeting. Varasteh and co-workers reported that the ^68^Ga-labeled nanobody ^68^Ga-NOTA-anti-MMR Nb showed promising results for the detection of myocardial infarction and atherosclerotic lesions [8, 9]. ^68^Ga-labeled mannosylated human serum albumin (^68^Ga-NOTA-MSA) has also shown promising results for PET imaging of sentinel lymph nodes [11] and assessment of pulmonary arterial hypertension-induced inflammation in lungs [12]. The ^18^F- and ^68^Ga-radiolabeled camelid single-domain antibody fragments [^18^F]FB-anti-MMR-sdAb and [^68^Ga]Ga-NOTA-anti-MMR-sdAb are able to detect CD206-positive tumor-associated macrophages, and the latter has recently entered in clinical studies [22–24]. Recently, a [^68^Ga]RP832c peptide has also been evaluated for PET imaging of tumor-associated macrophages [13]. Most of these tracers have utilized straightforward chelator-based radiolabeling. The use of a ^68^Ge/^68^Ga generator simplifies the production process of ^68^Ga-labelled tracers. However, compared to ^68^Ga, ^18^F offers better spatial resolution for PET images due to its ideal physical properties. In general, antibody/antibody fragments-based tracers have better targeting specificity, but as a direct comparison between CD206-targeted tracers is still lacking, this remains to be investigated in the future. Al[^18^F]F-NOTA-D10CM and ^68^Ga-NOTA-MSA recognize the carbohydrate-rich domain (CRD) of CD206 via their mannose structure, while ^68^Ga-NOTA-anti-MMR Nb, [^18^F]FB-anti-MMR-sdAb, and [^68^Ga]Ga-anti-CD206-sdAb recognize specific protein sequences outside the CRD. The highest uptake of mannosylated ligands occurs in the liver, followed by the spleen and bone marrow, reflecting the presence of CD206-positive cells, and excess radioactivity is excreted via the kidneys into the urinary bladder. Tracers based on antibody fragments or peptides show the highest uptake in the renal cortex, which is typical of smaller compounds, followed by the liver and spleen. Overall, both mannosylated and MMR-antibody fragments-based tracers exhibit excellent in vivo stability. The Al[^18^F]NOTA-DC10M shows fast excretion kinetics as expected due to its chemical structure. If the tracer would be translated into clinical use, similar kinetics to [^18^F]FDG would be expected. In addition to imaging inflammation by i.v. injection, our mannosylated dextran derivative also showed potential for imaging lymphatics and activated lymph nodes by i.d. administration, which is an advantage over described other tracers.

### Study limitations

We acknowledge that our study has some limitations. Only female mice were studied for CFA-induced inflammation. It is known that gender influences immune responses. Females often mount stronger innate and adaptive immune responses, including higher antibody responses to vaccinations, and have higher incidence of several autoimmune diseases [25]. Based on our previous experience with inflammation models and the increasing knowledge on differences in male and female immunity, we decided to use only female mice for the inflammation studies to avoid gender-induced variation to the study. In the future, Al[^18^F]F-NOTA-D10CM uptake in inflammation should also be investigated in males. Another study limitation appears in the in vitro binding study. Dose-dependent competition/blocking or use of another CD206-specific compound with a different structure would better confirm binding specificity. In our previous study, we performed an in vivo blocking study in healthy Sprague–Dawley rats using another CD206 targeting compound, mannan, to demonstrate significantly lower uptake of Al[^18^F]F-NOTA-D10CM in CD206-rich tissues such as liver, spleen and bone marrow [14]. In the future, we may consider the use of mannan in blocking studies of inflammation models. In addition, it can be regarded as a limitation that we did not perform ex vivo gamma counting and digital autoradiography of inflamed and contralateral joints. In practice this is very difficult to implement considering the small anatomical structures (synovial membrane) and the relatively short physical half-life of the ^18^F radionuclide.

## Conclusion

Our results support the conclusion that the mannosylated dextran derivative Al[^18^F]F-NOTA-D10CM specifically detects macrophage mannose receptor CD206 in inflamed tissue and draining lymph nodes. Further studies in a translational animal model with an inflammatory disease are warranted.

### Supplementary information

Below is the link to the electronic supplementary material.

Supplementary file1 (PDF 618 KB) file has been corrected

## Data Availability

The original data of the work can be obtained from Prof. Anne Roivainen upon rational request.
