# Supplementary material for: Macrophage mannose receptor CD206 targeting of fluoride-18 labeled mannosylated dextran: A validation study in mice
Source: Eur J Nucl Med Mol Imaging. 2024 Mar 27;51(8):2216–28. doi: 10.1007/s00259-024-06686-x (PMC11178572; doi:10.1007/s00259-024-06686-x)
Supplement: Supplementary file 1 — Supplementary file1 (PDF 618 KB) file has been corrected [file 259_2024_6686_MOESM1_ESM.pdf]

Supplementary material

Macrophage mannose receptor CD206 targeting of fluoride-18 labeled mannosylated dextran: A validation study in mice

Putri Andriana[1], Ruth Fair-Mäkelä[2,3], Heidi Liljenbäck[1,4], Salli Kärnä[1], Imran Iqbal[1], Konstantina Makrypidi[5], Johan Rajander[6], Ioannis Pirmettis[5], Xiang-Guo Li[1,3,7], Sirpa Jalkanen[8,3], Antti Saraste[1,9,10], Marko Salmi[2,3,8], and Anne Roivainen[1,3,4,9]

[1]Turku PET Centre, University of Turku, Turku, Finland; [2]Institute of Biomedicine, University of Turku, Turku, Finland; [3]InFLAMES Research Flagship Center, University of Turku, Turku, Finland; [4]Turku Center of Disease Modeling, University of Turku, Turku, Finland; [5]Institute of Nuclear and Radiological Science and Technology, Energy and Safety, NCSR "Demokritos", Athens, Greece; [6]Turku PET Centre, Accelerator Laboratory, Åbo Akademi University, Turku, Finland; [7]Department of Chemistry, University of Turku, Turku, Finland; [8]MediCity Research Laboratory, University of Turku, Turku, Finland; [9]Turku PET Centre, Turku University Hospital, Turku, Finland; and [10]Heart Center, Turku University Hospital and University of Turku, Turku, Finland

Correspondence: Professor Anne Roivainen, PhD, Turku PET Centre, Kiinamyllynkatu 4-8, FI-20520 Turku, Finland; Phone: +35823132862; E-mail: anne.roivainen@utu.fi

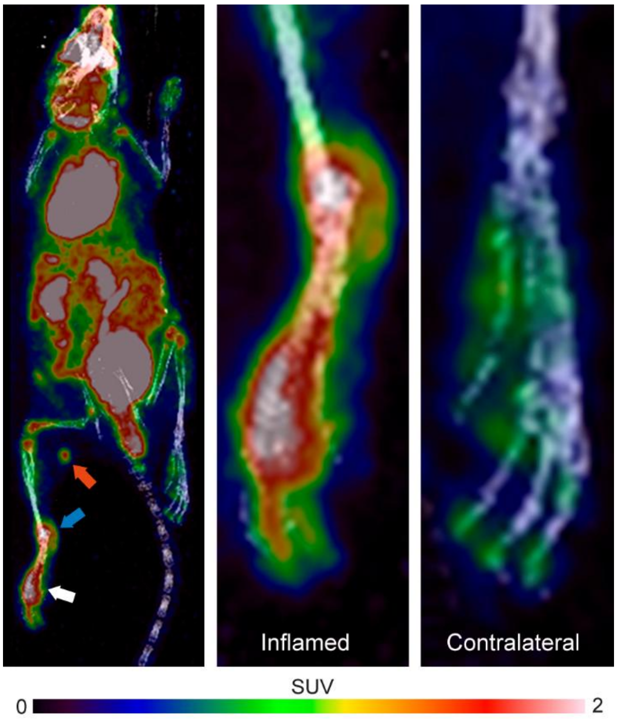

Supplementary Fig 1 [^{18}F]FDG PET/CT of a mouse with complete Freund's adjuvant-induced inflammation shows high uptake in the left inflamed popliteal lymph node (red arrow), ankle joint (blue arrow), and foot pad skin (white arrow)

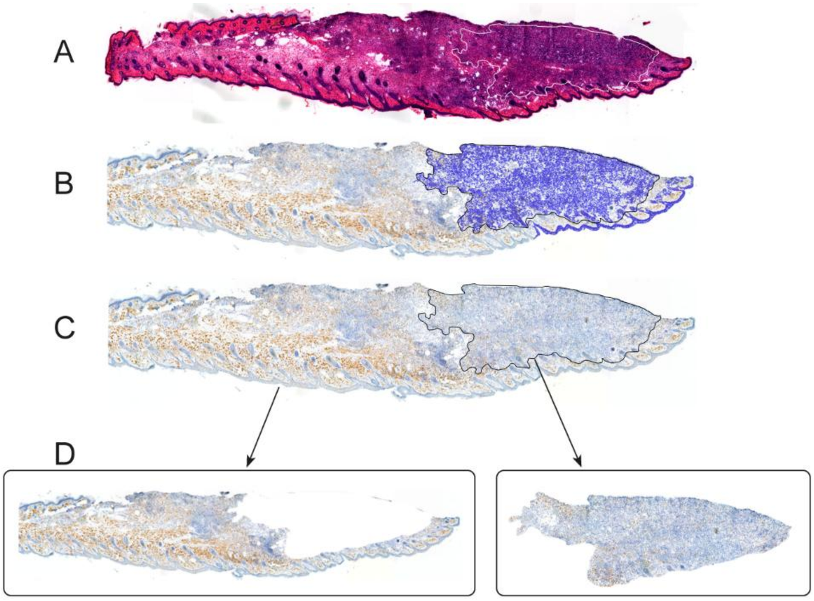

Supplementary Fig 2 Histology and immunohistochemical staining of foot pad skin. (A) H&E staining reveals that the complete Freund's adjuvant subcutaneously injected on the dorsal side of the left hind paw has inflamed the skin. (B) Regions with low CD206 ($CD206_{low}$) were visually observed in less inflamed areas according to H&E stain, and these were classified as $CD206_{low}$ areas using fuzzy tools in GIMP based on an RGB color threshold of 19 and color inversion to show a clear boundary of the $CD206_{low}$ area. The contours were delineated based on the clear-cut boundary and reproduced on the original CD206-stained section. The $CD206_{low}$ area (C, right panel) is separated from the $CD206_{high}$ area (C, left panel) following the contour, and the DAB staining was analyzed for color deconvolution using Fiji image processor software from ImageJ

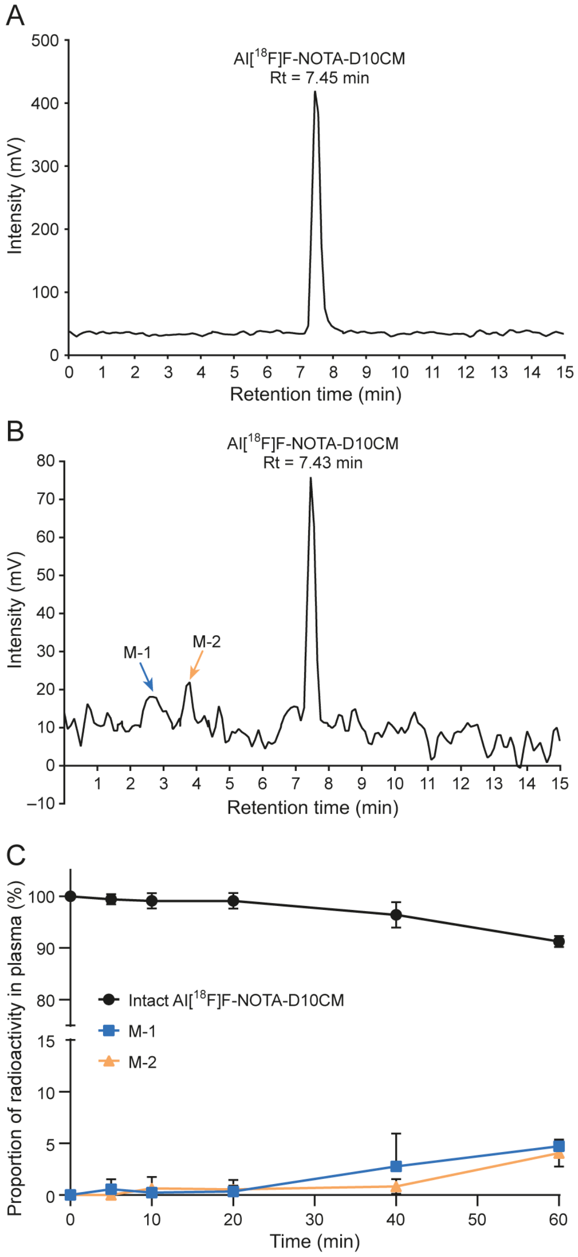

Supplementary Fig. 3 *In vivo* stability of Al[^{18}F]F-NOTA-D10CM in healthy C57BL/6N mice. Representative radio-HPLC chromatograms of (A) intact Al[^{18}F]F-NOTA-D10CM and (B) a plasma sample obtained at 60 minutes after intravenous injection. (C) Percentage of intact Al[^{18}F]F-NOTA-D10CM in plasma at different time points after injection. The radiometabolites M-1 and M-2 were not identified

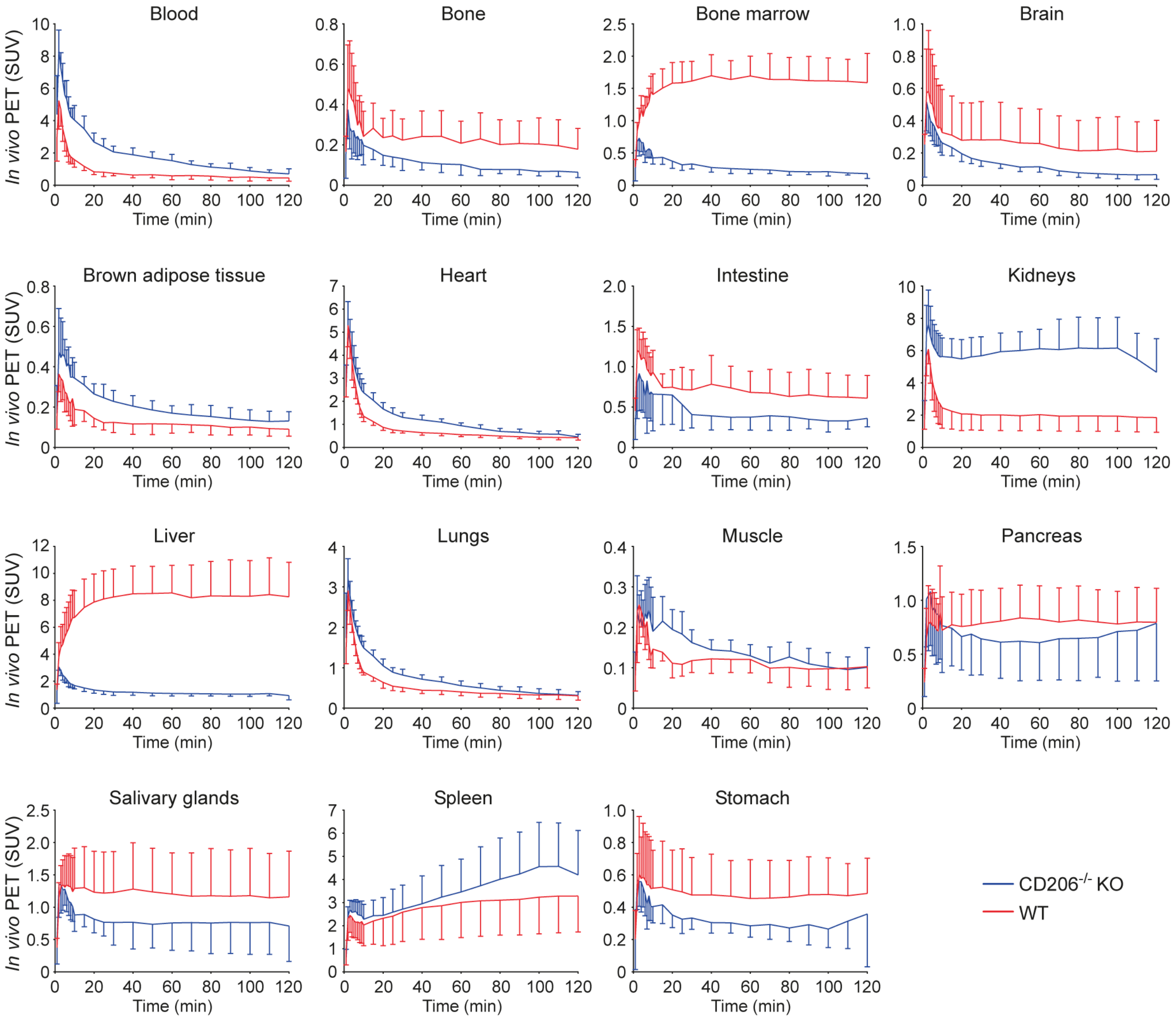

Supplementary Fig 4 Time-activity curves of Al[^{18}F]F-NOTA-D10CM in wild-type (WT) and CD206$^{-/-}$ knockout (KO) mice. The lines are standardized uptake value (SUV) and error bars are standard deviation. WT, *n* = 7; CD206$^{-/-}$ KO, *n* = 8

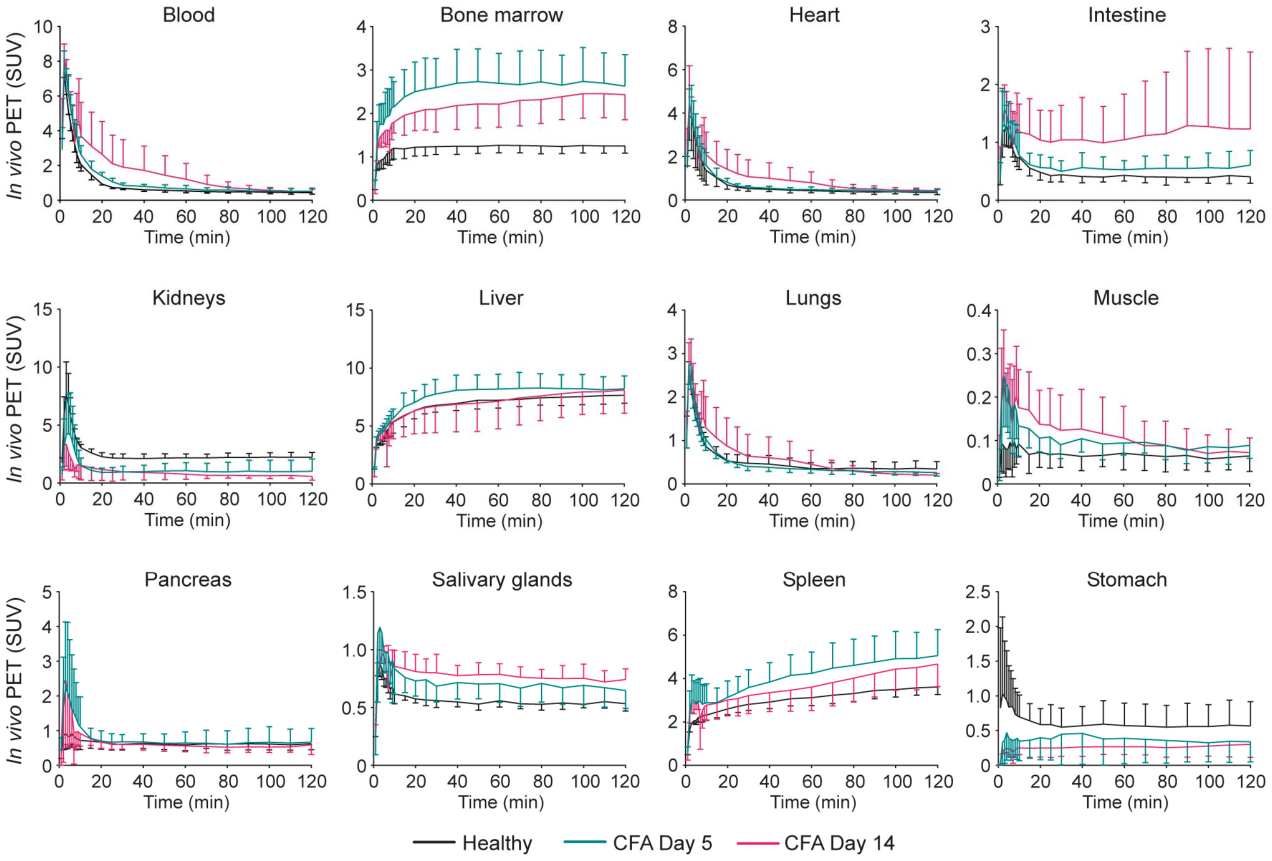

Supplementary Fig 5 Regional time-activity curves of intravenously injected Al[^{18}F]F-NOTA-D10CM in inflamed and healthy mice. Lines represent mean and error bars are standard deviation (healthy, *n* = 4; CFA day 5, *n* = 6; CFA day 14, *n* = 10). CFA, complete Freund's adjuvant

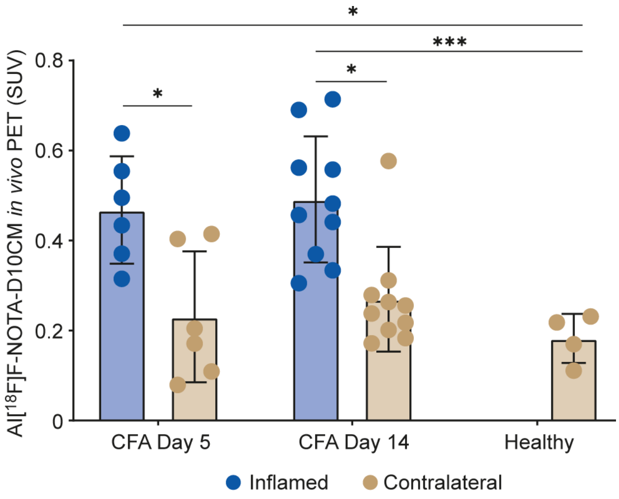

Supplementary Fig 6 *In vivo* uptake of Al[^{18}F]F-NOTA-D10CM in ankle joints of complete Freund's adjuvant (CFA)-induced inflammation mice compared with healthy mice. Standardized uptake values (SUV) were extracted from 50–60 minute time frames after injection and are expressed as mean ± SD. *P<0.05, ***P<0.001

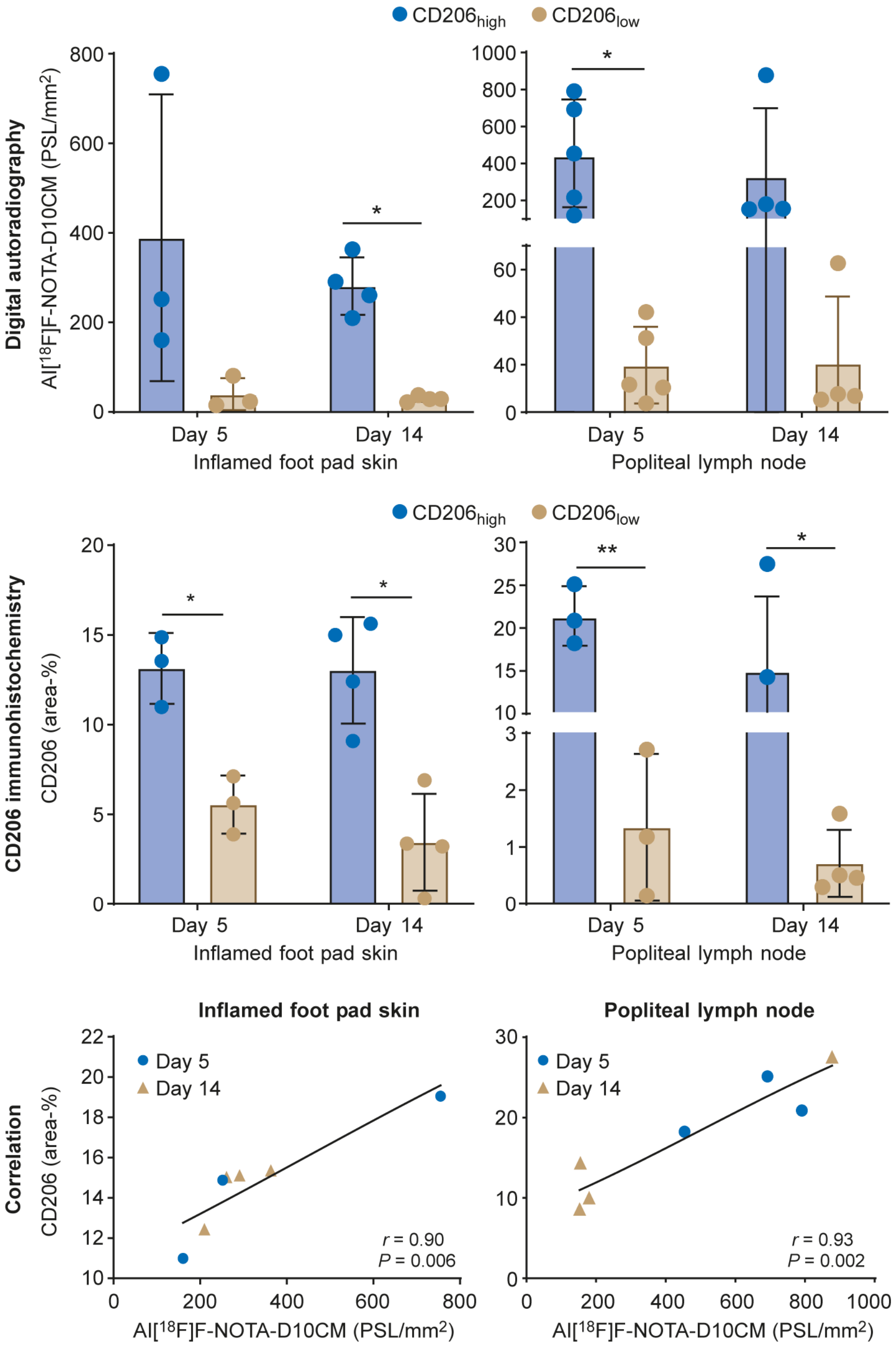

Supplementary Fig 7 Quantification of autoradiographs following intravenous injection of Al[^{18}F]F-NOTA-D10CM and CD206 immunohistochemical staining of inflamed skin and inflamed lymph node on day 5 and day 14 after CFA-induced inflammation, and correlation of Al[^{18}F]F-NOTA-D10CM uptake with CD206-positivity. **P*<0.05, ***P*<0.01. PSL, photostimulated luminescence

Supplementary Table 1 *Ex vivo* biodistribution of Al[^{18}F]F-NOTA-D10CM at 60 minutes after intravenous injection in WT and CD206$^{-/-}$ KO mice

Tissue	WT<br>(*n* = 7)	CD206$^{-/-}$ KO<br>(*n* = 8)	*P* value
Blood	0.37 ± 0.20	2.77 ± 0.83	<0.001
Bone (skull)	1.68 ± 0.40	0.51 ± 0.12	0.001
Bone marrow (femur)	19.63 ± 2.76	1.00 ± 0.51	<0.0001
Heart	2.01 ± 0.52	0.81 ± 0.36	<0.001
Intestine (large)	4.30 ± 0.73	1.05 ± 0.48	<0.0001
Intestine (small)	4.30 ± 0.73	1.25 ± 0.52	<0.0001
Kidneys	8.95 ± 5.20	32.03 ± 13.83	0.002
Liver	40.18 ± 11.21	4.56 ± 1.74	<0.001
Lungs	0.54 ± 0.14	1.32 ± 0.74	0.017
Muscle	0.26 ± 0.10	0.27 ± 0.10	0.586
Pancreas	2.82 ± 0.43	1.05 ± 0.42	<0.001
Salivary glands	4.64 ± 1.90	1.40 ± 0.34	0.011
Spleen	25.15 ± 6.78	43.71 ± 16.49	0.045
Stomach	0.77 ± 0.36	0.64 ± 0.54	0.435

Results are expressed as percentage of injected radioactivity dose per gram of tissue (mean ± SD)

Supplementary Table 2 *Ex vivo* biodistribution of Al[^{18}F]F-NOTA-D10CM at 60 minutes after intravenous injection in inflamed and healthy mice

Tissue	CFA-induced inflammation			Healthy	*P*-values
	Day 7 (n = 5)	Day 16 (n = 6)	Pooled (n = 11)	(n = 4)	
Blood	0.75 ± 0.37	0.60 ± 0.12	0.61 ± 0.20	1.07 ± 0.30	0.043
Bone (skull)	1.91 ± 0.43	2.45 ± 0.37	2.15 ± 0.48	1.87 ± 0.19	0.170
Bone marrow (femur)	39.32 ± 14.90	62.52 ± 22.62	51.46 ± 19.40	42.11 ± 7.52	0.288
Bone + bone marrow (femur)	8.55 ± 2.58	8.65 ± 1.92	8.60 ± 2.10	10.82 ± 0.51	0.021
Heart	3.62 ± 1.57	3.29 ± 0.33	3.47 ± 1.14	4.09 ± 0.43	0.187
Intestine (large)	4.88 ± 2.64	4.03 ± 0.31	4.50 ± 1.93	5.34 ± 0.29	0.238
Intestine (small)	5.04 ± 2.65	8.72 ± 0.32	6.88 ± 2.63	9.23 ± 0.78	0.045
Kidneys	2.74 ± 0.63	2.65 ± 0.64	2.70 ± 0.59	19.04 ± 3.49	0.002
Liver	42.18 ± 12.19	35.56 ± 7.58	38.87 ± 10.04	42.56 ± 6.16	0.603
Lymph node (popliteal, left)	1.59 ± 0.96	1.00 ± 0.22	1.34 ± 0.76	ND	ND
Lungs	0.81 ± 0.11	0.72 ± 0.23	0.77 ± 0.17	1.27 ± 0.32	0.044
Muscle (thigh, left)	3.83 ± 0.18	3.16 ± 0.21	3.45 ± 0.40	0.26 ± 0.04	<0.0001
Muscle (thigh, right)	0.39 ± 0.28	0.34 ± 0.18	0.36 ± 0.23	ND	ND
Pancreas	1.64 ± 1.08	3.01 ± 0.05	3.55 ± 1.22	3.62 ± 0.16	0.866
Salivary gland	2.80 ± 0.70	2.59 ± 0.61	2.71 ± 0.63	3.46 ± 0.78	0.153
Skin (footpad, right)	0.78 ± 0.17	0.66 ± 0.28	0.72 ± 0.22	ND	ND
Skin (footpad, left)	1.46 ± 0.59	1.43 ± 0.31	1.44 ± 0.44	0.74 ± 0.21	0.002
Spleen	47.23 ± 7.34	34.69 ± 6.27	38.86 ± 11.29	27.79 ± 6.04	0.063
Stomach	1.94 ± 0.95	1.65 ± 0.51	1.81 ± 0.76	2.01 ± 0.54	0.599

Results are expressed as percentage of injected radioactivity dose per gram of tissue (mean ± SD). ND, not determined

Supplementary Table 3 *Ex vivo* biodistribution of Al[^{18}F]F-NOTA-D10CM at 60 minutes after intradermal versus intravenous injection in inflamed mice

Tissue	Day 16 after CFA-induced inflammation		*P*-values
	Intradermal (*n* = 4)	Intravenous (*n* = 6)	
Blood	0.26 ± 0.05	0.60 ± 0.12	0.006
Bone (skull)	0.55 ± 0.07	2.45 ± 0.37	0.001
Bone marrow (both femur)	7.28 ± 0.56	62.52 ± 22.62	0.006
Bone + bone marrow (femur)	1.76 ± 0.31	8.65 ± 1.92	0.006
Heart	0.61 ± 0.15	3.29 ± 0.33	<0.0001
Intestine (large)	1.29 ± 0.24	4.03 ± 0.31	<0.0001
Intestine (small)	1.68 ± 0.35	8.72 ± 0.32	<0.0001
Kidneys	1.03 ± 0.11	2.65 ± 0.64	0.014
Liver	19.59 ± 0.13	35.56 ± 7.58	0.024
Lymph node (popliteal, left)	68.82 ± 25.95	1.00 ± 0.22	0.014
Lymph node (popliteal, right)	1.49 ± 1.24	ND	ND
Lungs	0.24 ± 0.05	0.72 ± 0.23	0.024
Muscle (thigh, left)	0.13 ± 0.08	3.16 ± 0.21	<0.0001
Muscle (thigh, right)	0.07 ± 0.01	0.34 ± 0.18	0.065
Pancreas	0.84 ± 0.12	3.01 ± 0.05	<0.0001
Salivary gland	0.74 ± 0.12	2.59 ± 0.61	0.008
Skin (footpad, left)	71.37 ± 20.62	0.66 ± 0.28	0.028
Skin (footpad, right)	0.31 ± 0.11	1.43 ± 0.31	0.153
Spleen	11.15 ± 0.33	34.69 ± 6.27	0.013
Stomach	0.28 ± 0.08	1.65 ± 0.51	0.012

Results are expressed as percentage of injected radioactivity dose per gram of tissue (mean ± SD). ND, not determined
